# AmLexin, a Standardized blend of Acacia catechu and Morus alba, shows benefits to delayed onset muscle soreness in healthy runners

**DOI:** 10.20463/jenb.2018.0027

**Published:** 2018-12-31

**Authors:** Mesfin Yimam, Shawn M. Talbott, Julie A. Talbott, Lidia Brownell, Qi Jia

**Affiliations:** 1 Research and Development, Unigen Inc, Tacoma USA; 2 Human Clinical Trial, GLH Nutrition LLC, Draper USA

**Keywords:** Delayed onset muscle soreness, Acacia catechu, Morus alba, inflammation, oxidative stress

## Abstract

**[Purpose]:**

Sudden and exhaustive exercise causes muscle damage accompanied by oxidative stress and inflammation, leading to muscle fatigue and soreness. AmLexin contains a standardized blend of extracts from the heartwood of Acacia catechu and the root bark of Morus alba, and is known to possess antioxidant and anti-inflammatory properties. The aim of this study was to investigate the effects of this proprietary blend supplementation on muscular pain and redox balance in healthy runners, in comparison to a placebo.

**[Methods]:**

A double-blind placebo-controlled clinical trial was carried out over 9 weeks in a single center. Thirty physically active male and female subjects within 18−70 years of age were randomized into AmLexin (mean age = 42.92 ± 2.48 and gender 7/5, male/female, respectively) and placebo (mean age = 41.15 ± 3.5 and gender 10/3, male/female, respectively) groups. Subjects were supplemented with 400 mg of AmLexin/day or a look-alike placebo during an 8-week training program, and for one week following a 13.1-mile half-marathon. Twenty-six subjects completed the 9-week supplementation trial.

**[Results]:**

Results showed the AmLexin group experienced significantly lower levels of post-exercise pain on day 1−3 following the half-marathon compared to the placebo group. The AmLexin group also showed lower post-exercise oxidative stress and higher antioxidant capacity on days 1 and 6 following the half-marathon. These results demonstrated the rapid benefits of AmLexin on pain and oxidative stress within 1−6 days post-exercise.

**[Conclusion]:**

Our data suggest that AmLexin could be a safe, effective botanical alternative for delayed onset muscle soreness.

## INTRODUCTION

Individuals who are not accustomed to rigorous physical activity, or athletes who are returning to training from a prolonged period of inactivity, often experience a dull, aching pain that usually occurs within 24 hours after activity. This type of soreness is known as delayed onset muscle soreness (DOMS). It usually manifests within the first 24 hours after exercise, reaching its peak within 72 hours, and slowly resolves in 5–7 days post-exercise^1,2^. It presents clinically as discomfort, aching pain, swelling, tenderness, stiffness, and temporary loss of muscle strength and function^3,4^. Aggregated effects of DOMS could also lead to detrimental injuries. 

Etiological factors for DOMS involve both mechanical and biochemical components^5^. Among the multiple causes of DOMS sequential events, the following processes remain vital in the path to understanding this phenomenon: the initial damage, related to the mechanical stress of contraction that produce ruptures of myofibril filaments, and the secondary damage, related to later events of metabolic origin resulting loss of the intracellular calcium homeostasis, oxidative stress, and inflammation^6^. When initial eccentric damage to myofibrils occurs, there is an immediate decrease in muscle strength and membrane damage^7^. Damaged muscle fibers release prostaglandins and proinflammatory cytokines that activate endothelial cells. In turn, these cells express cell surface adhesion molecules and release chemotactic factors and proinflammatory cytokines (e.g., IL-1β, TNF-α, IL-6)^8^. These inflammatory mediators attract phagocytic cells such as neutrophils and monocytes toward the injury site. As a result, in the first few hours after injury, neutrophils and macrophages progressively accumulate in the subjected muscles, helping to remove and degrade damaged tissue by engulfing cellular debris and releasing proteases, inflammatory cytokines, and reactive oxygen species (ROS)^9^. ROS may further lead to activation of NF-κB that can mediate the expression of inflammatory cytokines. This may induce further inflammation and oxidative stress, leading to secondary muscle damage. In these orchestrated sequences of events, some factors seem to play important roles in muscle regeneration and remodeling^8-10^. When the excess mechanical and biochemical stress produced at the time of exercise overcome the antioxidant and anti-inflammatory capacity of the body, there will be sustained inflammation and oxidative stress, which ultimately lead to muscle injury, fatigue, and DOMS^10^. Given these facts, a natural product with known anti-inflammatory and antioxidant activity with a good record of accomplishment of safety may provide a better intervention to DOMS. 

Catechin, the major flavan in *Acacia catechu*, and the prenylated flavonoids and stilbenoids from the root bark of *Morus alba* L., possess properties suggestive of benefits in DOMS. These reported activities include: (i) inhibition of the activities of cyclooxygenase-2 (COX-2), 5-lipoxygenase (5-LOX), platelets phospholipase A2, and proinflammatory cytokines such as TNF-α, ILs 1, 2, 6, 8, and 12^11,12^ as a result of catechin; (ii) inhibition of inflammation activity^13^; (iii) suppression effect of T-cell migration and inflammation induction^14^; (iv) inhibition of nitrogen oxide (NO), inducible NO synthase expression, prostaglandin E2 production, and activation of NF-κB^15^; (v) inhibition of proinflammatory mediators such as IL-1β, and IL-6 and COX-2^16^; and (vi) activation of total antioxidant ability^17,18^ as a result of prenylated flavonoids and stilbenoids from *M. alba* root bark extract. 

Furthermore, the standardized blend AmLexin has showed anti-inflammatory and antioxidant properties when evaluated in our laboratory using multiple models. In the carrageenan-induced rat paw edema model, AmLexin resulted in 46.3–53.3% reductions in paw edema and 43.6–54.8% reductions in pain sensitivity (common cardinal signs of inflammation) compared to the vehicle-treated control^19^. IC_50_ (concentration causing 50% inhibition) values for major inflammation-mediating enzymes, such as cyclooxygenase 1 (COX-1), cyclooxygenase 2 (COX-2), and 5-lipoxygenase (5-LOX), were found to be 20.9 μg/mL, 49.2 μg/mL, and 11.1 μg/mL, respectively^19^. Administered at 50 mg/kg, AmLexin also showed statistically significant inhibition in key inflammatory cytokines such as IL-1β, TNF-α, and IL-6 in a collagen-induced rat arthritis model. AmLexin also showed higher capacity in neutralizing superoxide anions when tested for its antioxidant ability in an Oxygen Radical Absorption Capacity (ORAC) assay (manuscript submitted to Nutrients journal). 

Based on these anti-inflammatory and antioxidant properties of AmLexin, we hypothesized that AmLexin could be a safe and effective botanical alternative for DOMS. The current double-blind placebo-controlled clinical trial was therefore designed to evaluate this hypothesis. AmLexin is a proprietary combination of extracts from the heartwood of *Acacia catechu* and root bark of *Morus alba*.

## METHODS

### Composition

The Composition UP1306 (referred to as AmLexin^TM^) was prepared by mixing the standardized extract (that contains not less than 65% catechins) from the heartwood of *A. catechu *and *M. alba* root bark ethanol extract (that contains not less than 7% stilbenes and bioflavonoids) at a ratio of 1:2 by weight. The active contents in UP1306 are catechins (at least 15%), stilbenes, and bioflavonoids (at least 2%).

### Study design

**Figure 1.**
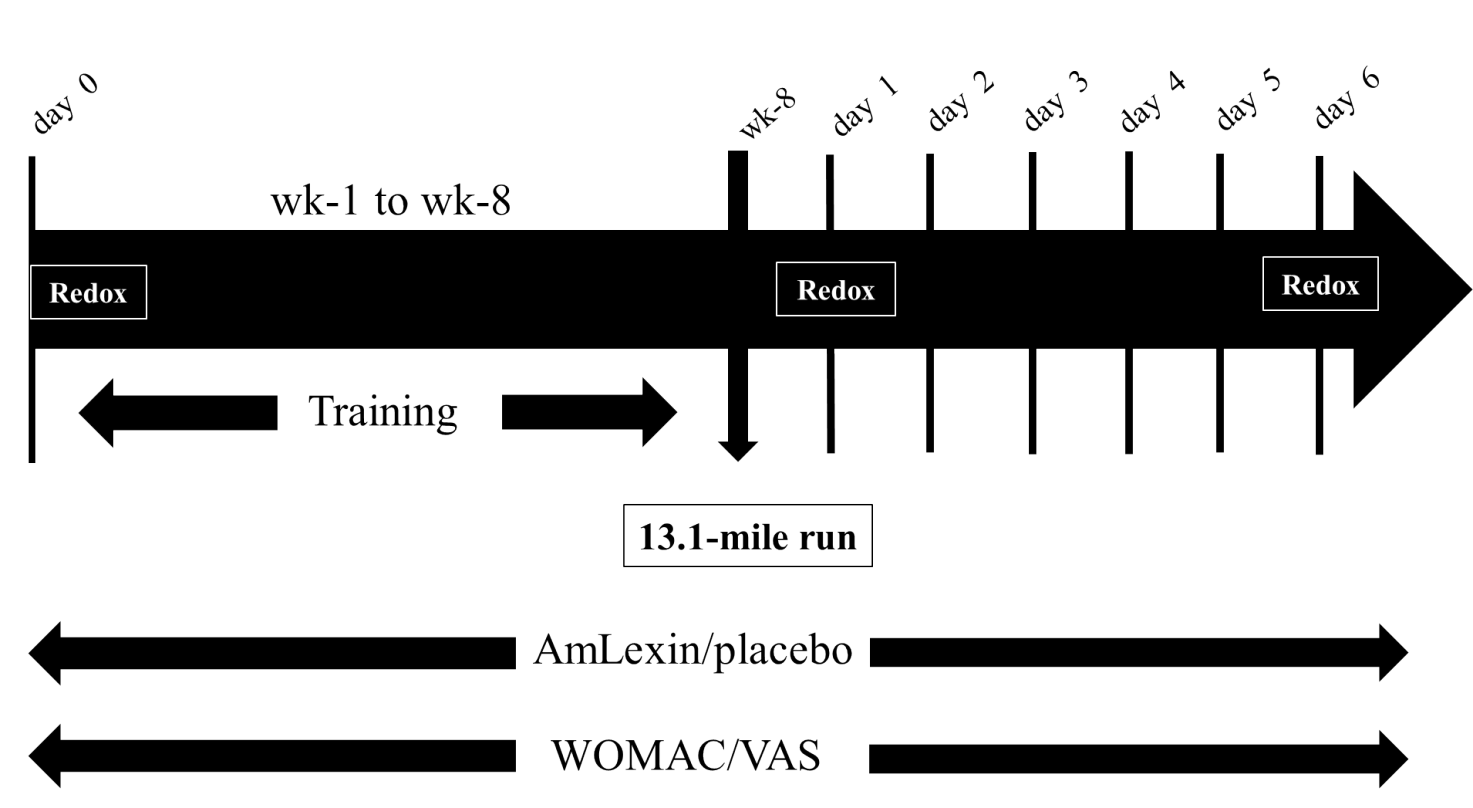
Clinical Trial Protocol

The study was a 9-week, prospective, randomized, double-blind, placebo-controlled parallel clinical trial conducted at a single study center, GLH Nutrition, Inc., Draper, UT. The study designs and protocols were approved by an IRB and informed consent was obtained from subjects. The primary goal of the study was to provide objective and subjective indicators demonstrating the ability of an herbal dietary supplement, AmLexin, to “protect joints” (proactively “prevent” pain and improve redox potential) in healthy subjects training for a 13.1 half-marathon run (non-disease claims). A total of 30 healthy subjects (15 per group; AmLexin/placebo) with age group 18–70 years (mean ± SE: 42.92 ± 2.48 and 41.15 ± 3.5 for AmLexin and placebo, respectively, and gender AmLexin = 7/5 and placebo 10/3 female/male, respectively) were enrolled in the study, and 25 subjects completed study participation (12 for AmLexin and 13 for placebo). Subjects were supplemented with AmLexin and the look-alike placebo for 9 weeks (8 weeks during training regimen and 1 week after the half-marathon run). The Western Ontario and McMaster University Osteoarthritis Index (WOMAC) for pain, stiffness, and activities of daily living, and the Visual Analog Scale for primary body sections/muscles (quadriceps, hamstrings, calves and knees) were taken at baseline (before starting the training program), weekly during the training regimen (weeks 1–8), and daily post-half-marathon (days 1–6). Blood was collected at baseline, day 1, and day 6 after exercise for oxidation-reduction potential monitoring (Fig. 1). 

### Product dosing

Subjects were instructed to take one capsule (200 mg of each) with a morning meal and one capsule (200 mg of each) with an evening meal, for a total of two capsules per day (total 400 mg/day AmLexin and Placebo). Subjects were instructed to bring product bottles to all visits. Product compliance was measured by pill counting of unused capsules at study completion.

### Subject inclusion criteria

• Males and females aged 18–70 years

• BMI < 30 kg/m^2^

• Healthy, active, no recurrent pain or limitations in range of motion

• Ability to complete training for and complete a 13.1-mile half-marathon run

• Willingness to consume the dietary supplements under investigation

• Willingness to undertake all protocol measurements and training regimens

• Ability to understand and sign the informed consent form (ICF)

### Subject exclusion criteria

• Recurring pain, use of pain medications or pain supplements

• Pregnant or breastfeeding women

• Inability to complete prescribed training regimen

• Current use of other “pain” support supplement or OTC drug

### Training program

On average, the weekly training regime consisted of subjects participating in a progressively more difficult 8-week running program to train for a hilly half-marathon run (13.1 miles). Each week, participants completed a minimum of three specific training runs of approximately 1 hour in duration, with progressively higher intensity and duration. Weekly runs included at least one “hill” workout, one “interval” workout, and one “distance” workout prescribed by the principal investigator.

### Informed consent

An informed consent form (ICF) was written and administered in accordance with established criteria of the Institutional Review Board (IRB) and the appropriate federal regulations (e.g., 21 CFR Parts 50 and 56) to describe the study plan, procedures, and risks. The investigator, the sponsor, and the IRB all approved the ICF. The study was reviewed by Aspire IRB, Santee CA 92071, with approval of protocol and documents for Unigen2017 received from IRB on 05/12/2017.

### Data management & analysis

Data were recorded onto case report forms (CRFs) by GLH Nutrition staff and entered into Excel worksheets. The computerized data were made anonymous, and subject identifiers were limited to two-digit ID numbers and subject initials. Data files are retained in a physically secure location, and regular backup copies are created and stored in a separate secure location. Prior to analysis, GLH Nutrition staff reviewed all data, compared the values in the data files with the values on the original CRFs, and corrected any discrepancies found. After unblinding, data was analyzed by the Per-Protocol (PP) method, consisting of all subjects who completed all scheduled visits with no protocol deviations. The primary efficacy endpoints were analyzed using SigmaPlot (version 11.0) between the product groups (active product or placebo). Statistical significance between groups was calculated by means of single factor analysis of variance, followed by a paired t-test. P-values less than or equal to 0.05 (P ≤ 0.05) were considered to be significant. When normality tests failed, data for nonparametric analysis were subjected to Mann-Whitney rank-sum tests and Kruskal-Wallis one-way analysis of variance on ranks for ANOVA.

### WOMAC

The Western Ontario and McMaster (WOMAC) index is a self-administered health status measure score based on 24 questions for three principal subscales, such as pain, stiffness, and activities of daily living^20^. The first five questions of WOMAC represent pain and measured from 0 (“no pain”) to 4 (“extreme pain”) for each question. This was followed by the stiffness subscale, based on the average of questions 6 and 7 and measured from 0 (“no stiffness”) to 4 (“extreme stiffness”) for each question. The rest of the questionnaire, questions 8 through 24 of the WOMAC, are allocated for activities of daily living and are measured from 0 (“no difficulty”) to 4 (“extreme difficulty”) for each question. The WOMAC is a valid, reliable, and sensitive instrument for the detection of clinically important changes in health status, following a variety of interventions such as AmLexin.

### Visual analog scale (VAS)

Measurements for delayed onset muscle soreness were assessed using visual analogue scale (VAS) score^21^. The pain VAS is a unidimensional measure of pain intensity, which has been widely used in diverse adult populations. The pain VAS is a continuous scale comprised of a horizontal (HVAS) or vertical (VVAS) line, usually 10 centimeters (100 mm) in length between “no pain” (score of 0) and “pain as bad as it could be” or “worst imaginable pain” (score of 100 [100-mm scale]). While questionnaires vary between study designs, most commonly, participants are asked to report “current” discomfort intensity and “in the last 24 hours.” In the current study, the VAS discomfort rating was used to record average discomfort with movement and at rest. VAS discomfort was rated from 0 (“no discomfort”) to 100 (“extreme discomfort”). The subjects answered the VAS questionnaire by placing a vertical line to indicate their intensity of discomfort relating to several body sections including quadriceps, calves, hamstrings, and knees. Using a ruler, the score is determined by measuring the distance (mm) on the 10 cm line between the “no pain” anchor and the patient's mark, providing a range of scores from 0–100. Data were analyzed to show the group mean values (mean ± SE), and the active (AmLexin) group was compared to the placebo. The average values were also normalized to 100, and percentage changes were calculated. Statistical significances were also calculated using a t-test. A higher average score value indicates greater discomfort intensity.

### Redox potential

The antioxidant activities of products were assessed using the RedoxSYS diagnostic system (Luoxis Diagnostics, Greenwood Village, CO, United States). The system measures the oxidation-reduction potential (ORP) of a biological system, displaying the degree of overall oxidative stress in response to exposure. The two primary components of the ORP, such as the static ORP (sORP) and the capacity ORP (cORP), are determined from plasma, and are measured in millivolts (mV) and microcoulombs (μC), respectively. While the low sORP values represent the normal range of oxidative stress (or less oxidative stress), the higher than normal sORP values indicate the biological sample is from an individual with a higher state of oxidative stress. The other segment of the ORP, cORP, is the measure of available antioxidant reserve. Higher cORP values correspond with better antioxidant reserves, and vice versa^22^.

## RESULTS

### WOMAC assessment for discomfort, stiffness and activities of daily living (ADL)

**Figure 2.**
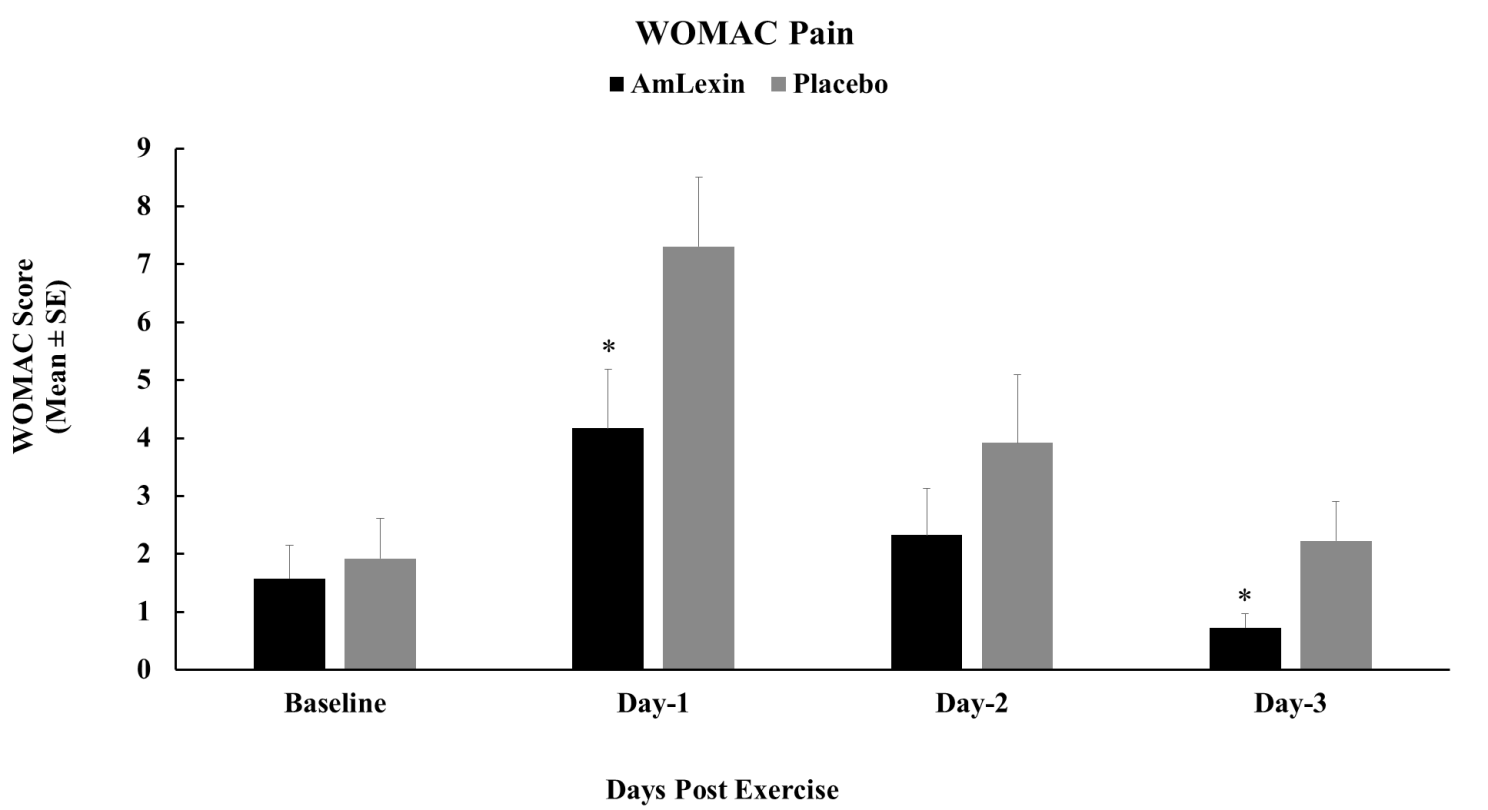
Effect of AmLexin on WOMAC pain. Healthy subjects were trained for 8-weeks and run a 13.1mile (half-marathon) on the 9^th^ week. Subjects were monitored for discomfort every day after the half-marathon. Data depicted as Mean ± SE. *P ≤ 0.05.

Subjects in the AmLexin group experienced significantly low levels of discomfort as of 24 hours after the half-marathon (Fig. 2). These reductions were maintained for the subsequent days after the run. When compared to subjects supplemented with the look-alike placebo, the improved comfort levels in the AmLexin group were statistically significant at days 1 (P < 0.05) and 3 (P < 0.03) (Fig. 2). These reductions in magnitude were compared between the groups, and were found to be 43%, 40.5%, and 67.4% reductions for the AmLexin group on days 1, 2, and 3, respectively. 

**Figure 3.**
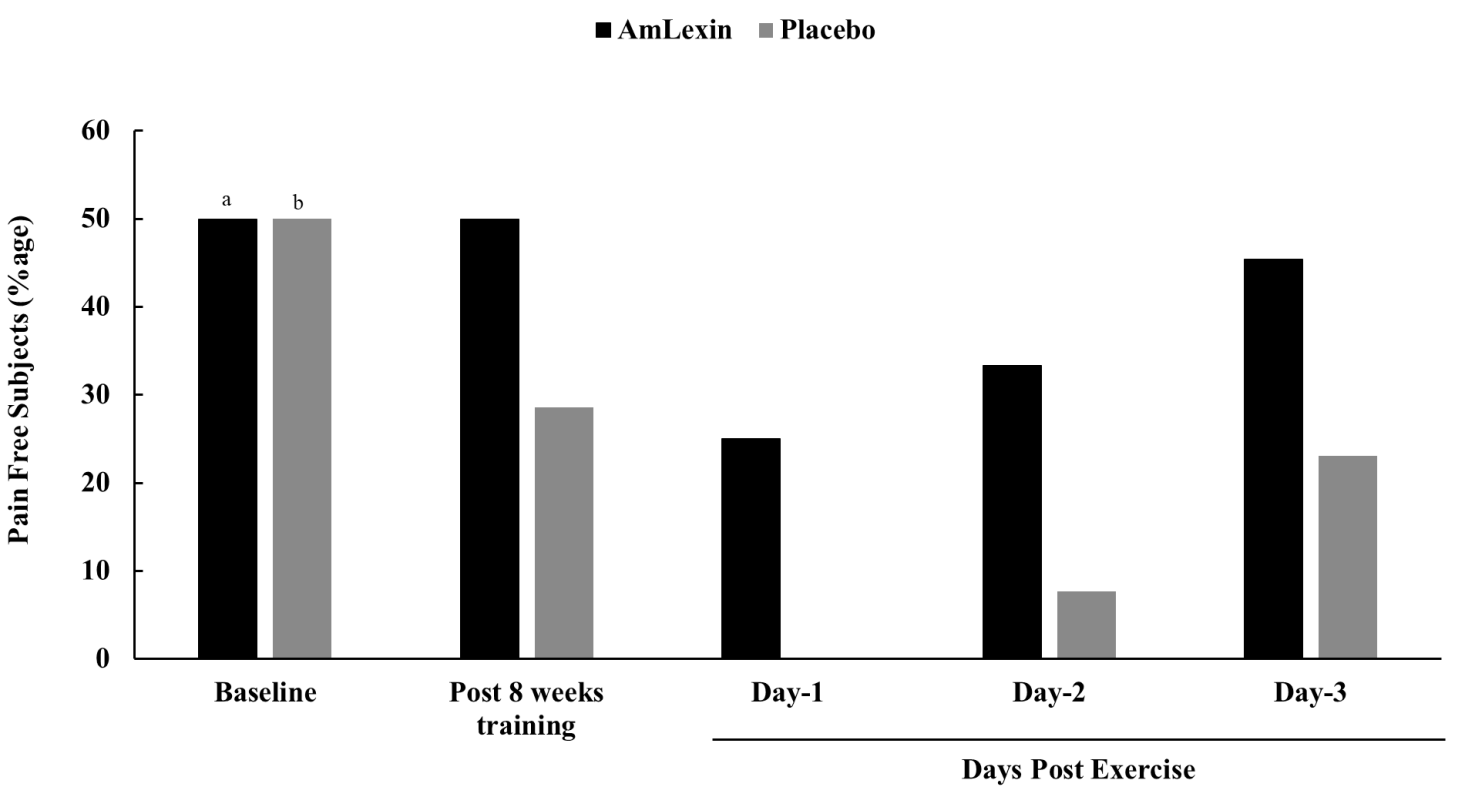
Percentage of subjects with no pain post exercise. Healthy subjects were trained for 8-weeks and run a 13.1mile (half-marathon) on the 9^th^ week. Subjects were monitored for discomfort every day after the half-marathon. ^*a*^* Ẋ=1.58±1.98; *^*b*^* Ẋ=1.36±1.86*

We also determined the percentage of subjects in each group with absolute “zero” discomfort (Fig. 3). While all subjects in the placebo group experienced some degree of discomfort, 25% of subjects in the AmLexin group had no symptoms at day 1 post-exercise. A higher percentage of subjects also reported minimal discomfort on days 2 (AmLexin = 33.3% and placebo = 7.7%) and 3 (AmLexin = 45.5% and placebo = 23.1%) after exercise compared with the placebo group, indicating the fast recovery of these subjects from DOMS (Fig. 3). 

**Figure 4.**
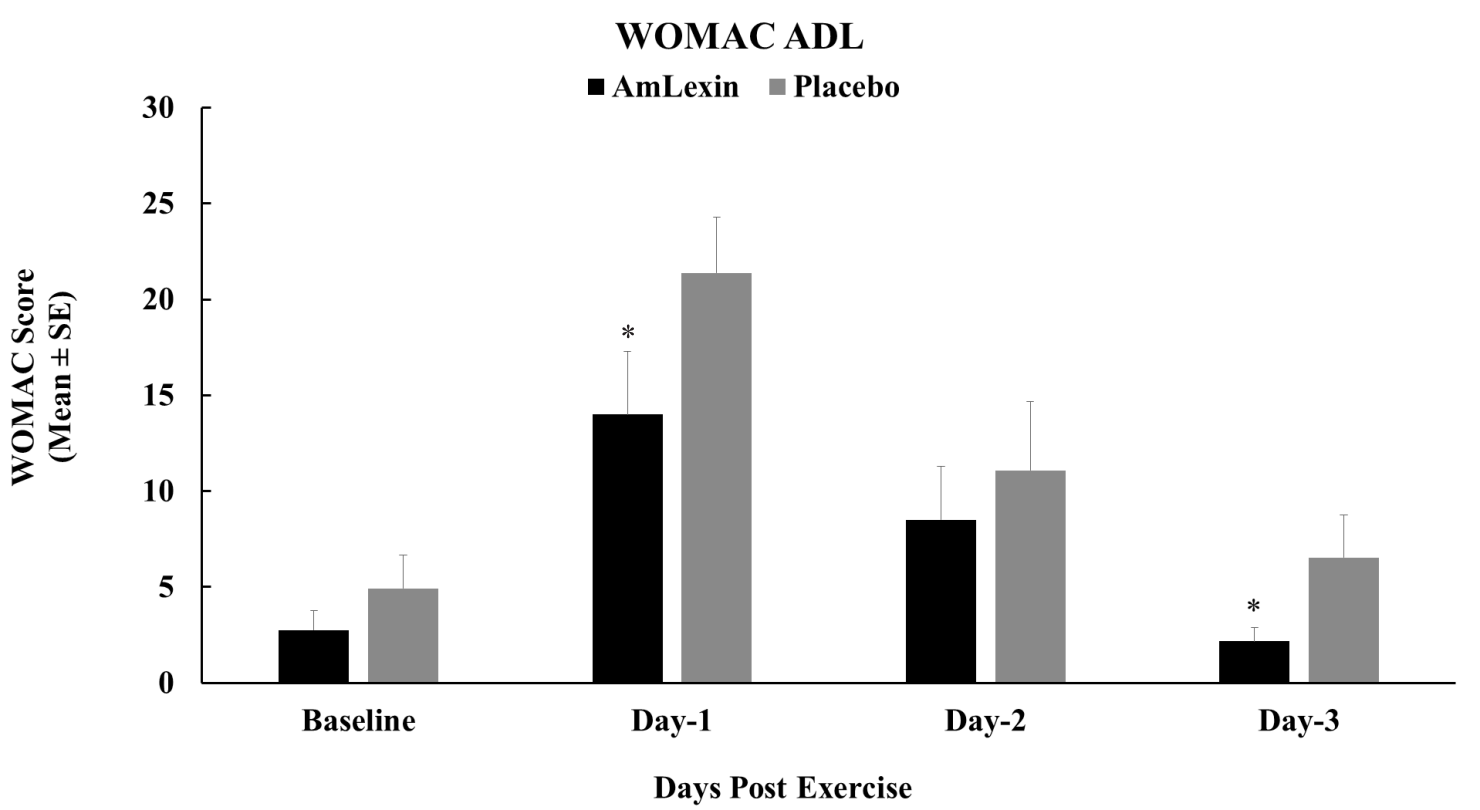
Effect of AmLexin on activities of daily living (ADL). Healthy subjects were trained for 8-weeks and run a 13.1mile (half-marathon) on the 9^th^ week. Subjects were monitored for their routine daily activity every day after the half-marathon. Data depicted as Mean ± SE. *P ≤ 0.05

Similar patterns were also observed in activities of daily living (ADL) (Fig. 4). Supplementation of AmLexin significantly improved the ADL of subjects as early as 24 hours post-exercise and remained low for the rest of the week. These improvements were statistically significant on days 1 (P < 0.05) and 3 (P < 0.05) post-exercise compared to the placebo group. With respect to magnitude, there were 34.5%, 23.3%, and 66.6% improvements in ADL compared to the placebo group after days 1, 2, and 3, respectively. 

**Figure 5.**
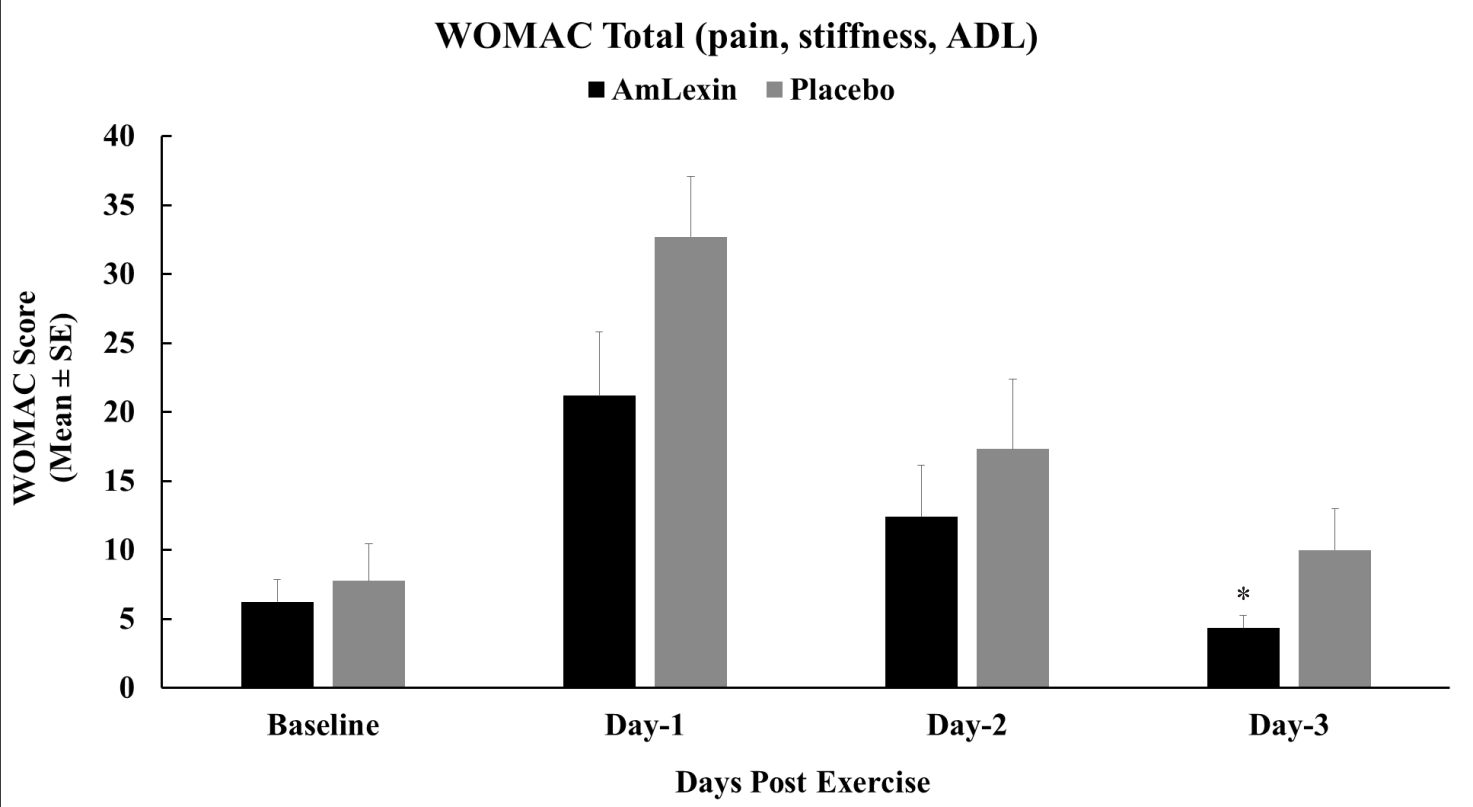
Effect of AmLexin on the overall total WOMAC. Healthy subjects were trained for 8-weeks and run a 13.1mile (half-marathon) on the 9^th^ week. Subjects were monitored every day after the half-marathon for discomfort, stiffness and ADL. Data depicted as Mean ± SE. *P ≤ 0.05.

While no significance was reported between groups for stiffness improvement, there were clear indications that subjects in the AmLexin group had lower stiffness than the placebo group (data not shown). Hence, the overall WOMAC for the discomfort, ADL, and stiffness were found within the ranges of the three subscales. The P-value on day 1 was not statistically significant for the total WOMAC (P = 0.08) (Fig. 5). Nevertheless, there were marked improvements in all parameters for the subjects supplemented with AmLexin with P ≤ 0.05 on day 3 (Fig. 5).

### Visual analog scale (VAS) of major muscle groups and body parts

**Figure 6.**
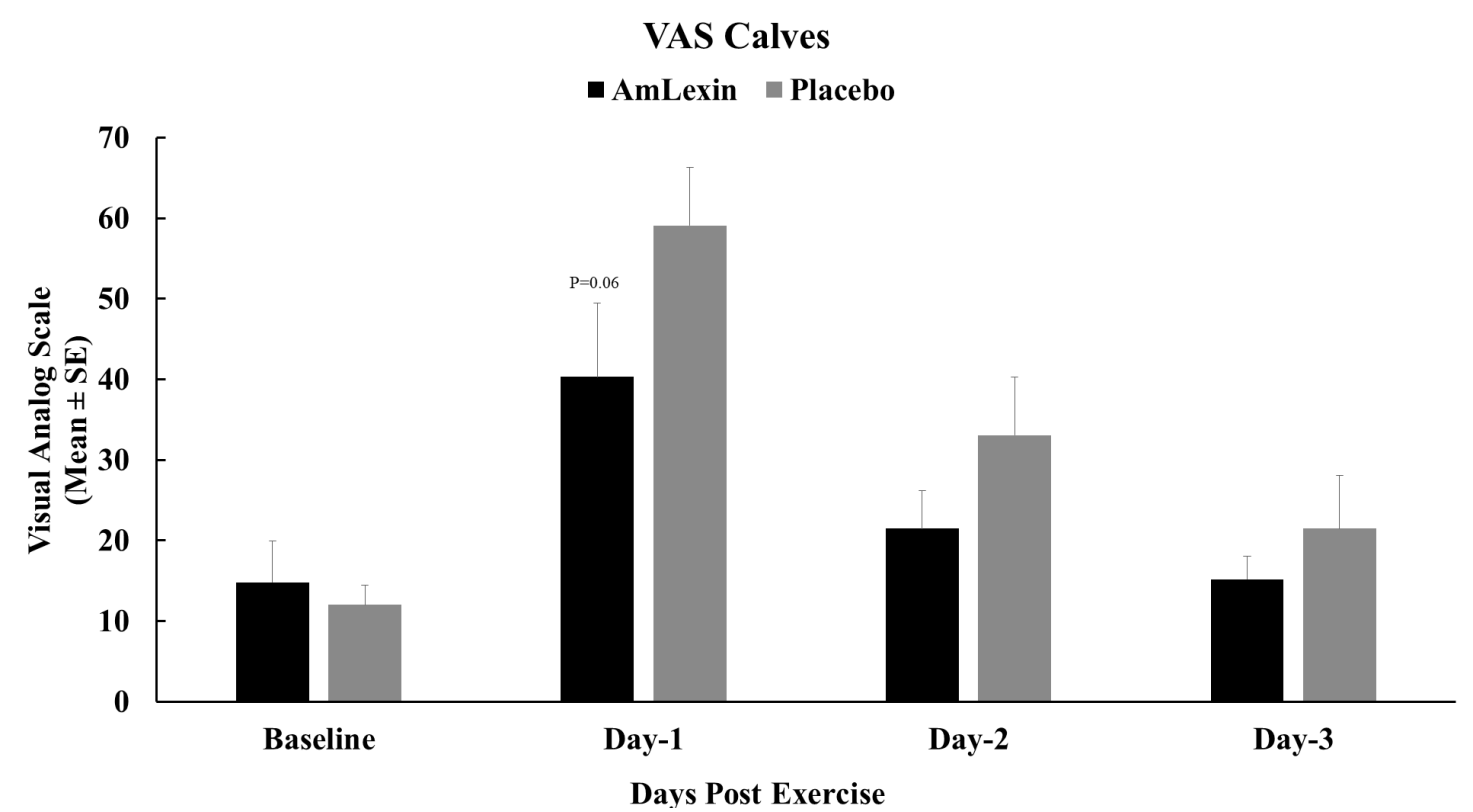
Effect of AmLexin on calf muscle VAS-discomfort. Healthy subjects were trained for 8-weeks and run a 13.1mile (half-marathon) on the 9^th^ week. Subjects were monitored every day after the half-marathon for discomfort on major muscles. Data depicted as Mean ± SE. P=0.06.

Complementing the WOMAC data above, subjects in the AmLexin group reported lesser discomfort over the course of the recovery time, as early as day 1 post-exercise (Fig. 6). Compared to the placebo treatment group, there were 31.8%, 35.0% and 29.3% reductions in discomfort from the calve muscles in the AmLexin treatment group, with a P-value of 0.06 on day 1 post-exercise (Fig. 6).

**Figure 7.**
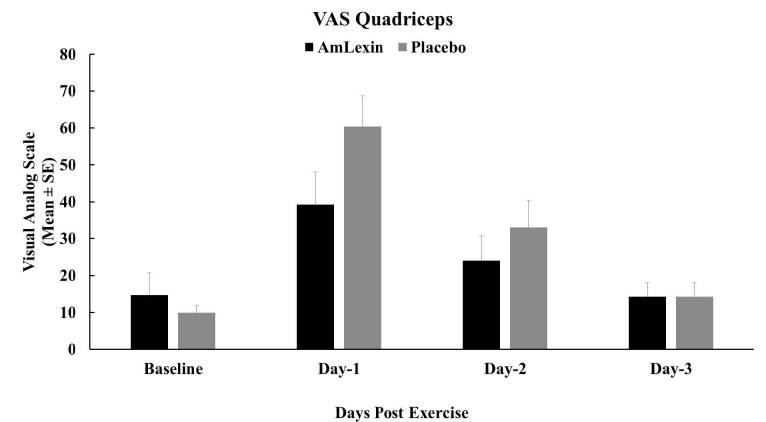
Effect of AmLexin on Quadriceps muscle VAS-discomfort. Healthy subjects were trained for 8-weeks and run a 13.1mile (half-marathon) on the 9^th^ week. Subjects were monitored every day after the half-marathon for discomfort on major muscles. Data depicted as Mean ± SE. *P ≤ 0.05.

Similarly, there were 34.9% and 27.3% reductions in VAS discomfort from the quadriceps of runners supplemented with AmLexin compared to the placebo on days 1 and 2 post-exercise. The relief in discomfort on day one after exercise was statistically significant (P < 0.05) when compared to the placebo group (Fig. 7).

**Figure 8.**
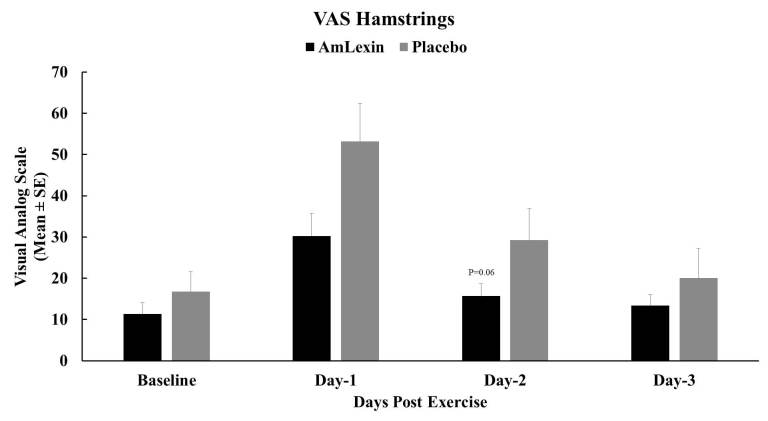
Effect of AmLexin on Hamstring muscle VAS-discomfort. Healthy subjects were trained for 8-weeks and run a 13.1mile (half-marathon) on the 9^th^ week. Subjects were monitored every day after the half-marathon for discomfort on major muscles. Data depicted as Mean ± SE. *P ≤ 0.05.

Perhaps the biggest discomfort relief was reported for the hamstrings of healthy runners supplemented with AmLexin (Fig. 8). Reductions of 43.2%, 46.4%, and 33.2% were observed on days 1, 2, and 3 after the half-marathon when compared to the placebo group. These reductions were statistically significant on day 1 (P < 0.05) with a similar trend on day 2 (P < 0.06) in comparison to the placebo group.

**Figure 9.**
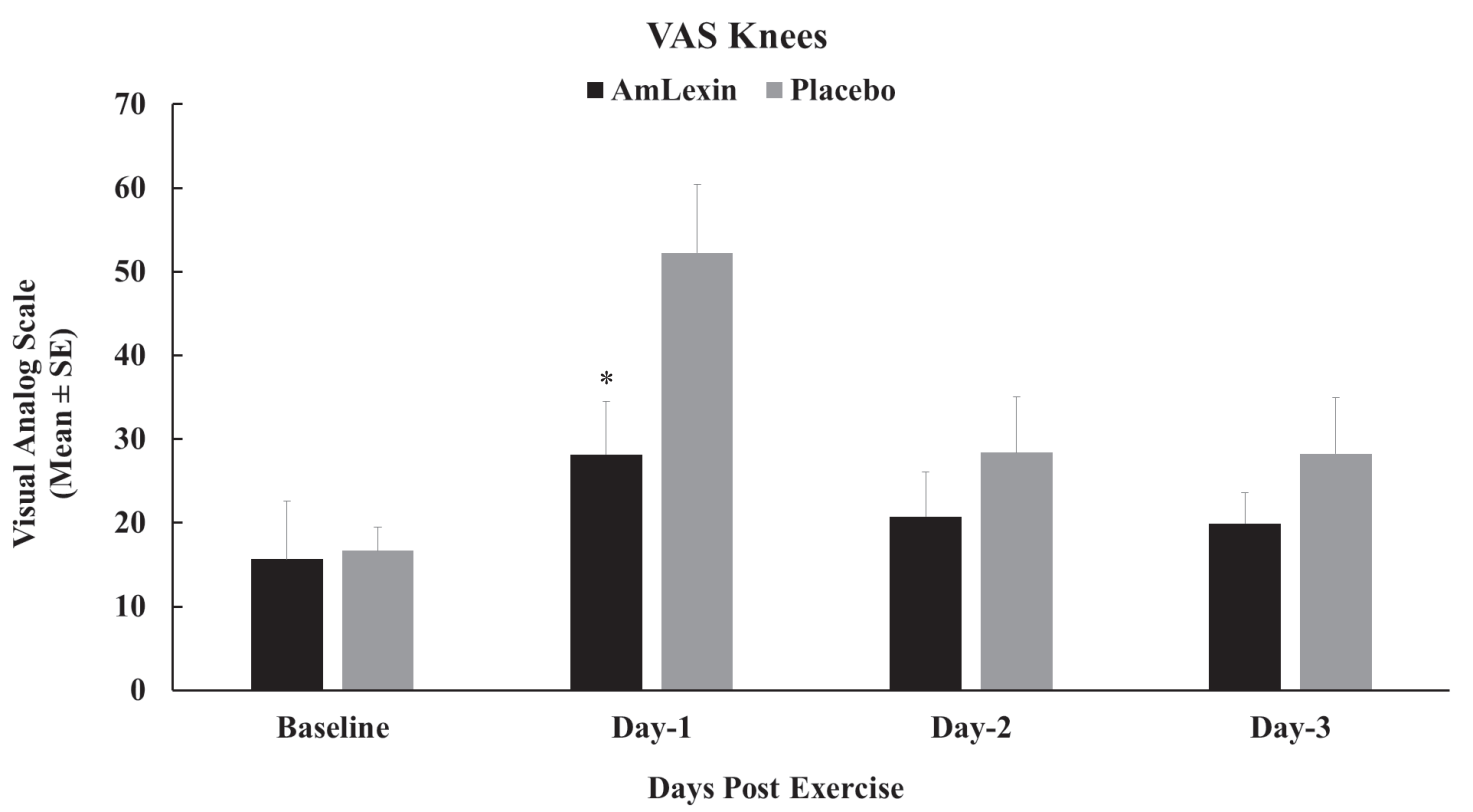
Effect of AmLexin on Knee VAS-discomfort. Healthy subjects were trained for 8-weeks and run a 13.1mile (half-marathon) on the 9^th^ week. Subjects were monitored every day after the half-marathon for discomfort on major muscles. Data depicted as Mean ± SE. *P ≤ 0.05.

Findings on the VAS in the knees were in accordance with the rest of the other muscle groups (Fig. 9). Subjects experienced minimal discomfort throughout the recovery period. There were 46.1%, 26.8%, and 29.4% reductions in discomfort for the subjects supplemented with AmLexin in comparison to the placebo group. The reduction observed on day one post exercise for the AmLexin group was statistically significant when compared to the placebo, at P < 0.05 (Fig. 9). 

### Antioxidant effect of test materials

**Figure 10.**
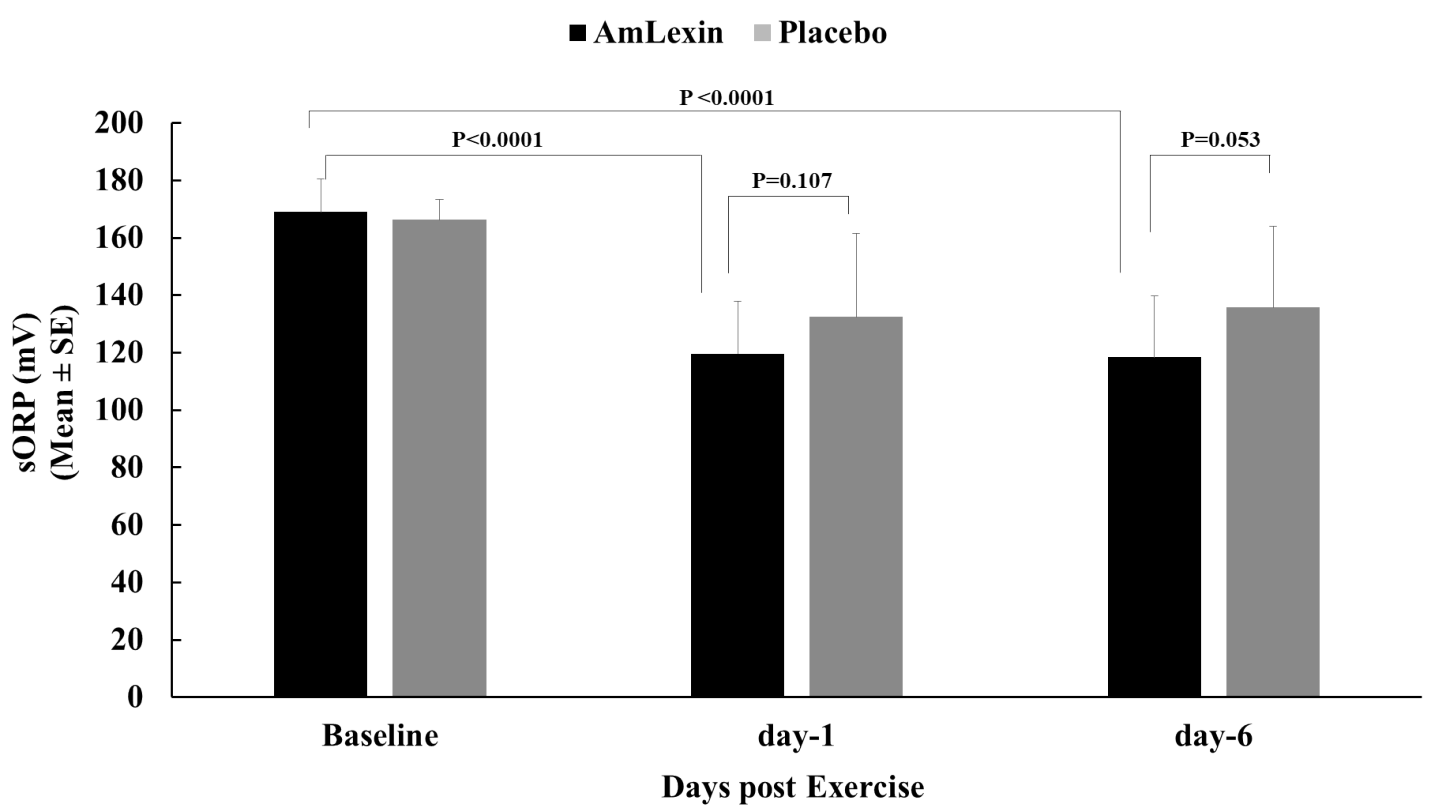
Effect of AmLexin on total body oxidative stress assessed using the RedoxSYS diagnostic system. Data are for static oxidation-reduction potential (sORP). Lower sORP indicates a better or reduced oxidative stress in the body.

**Figure 11.**
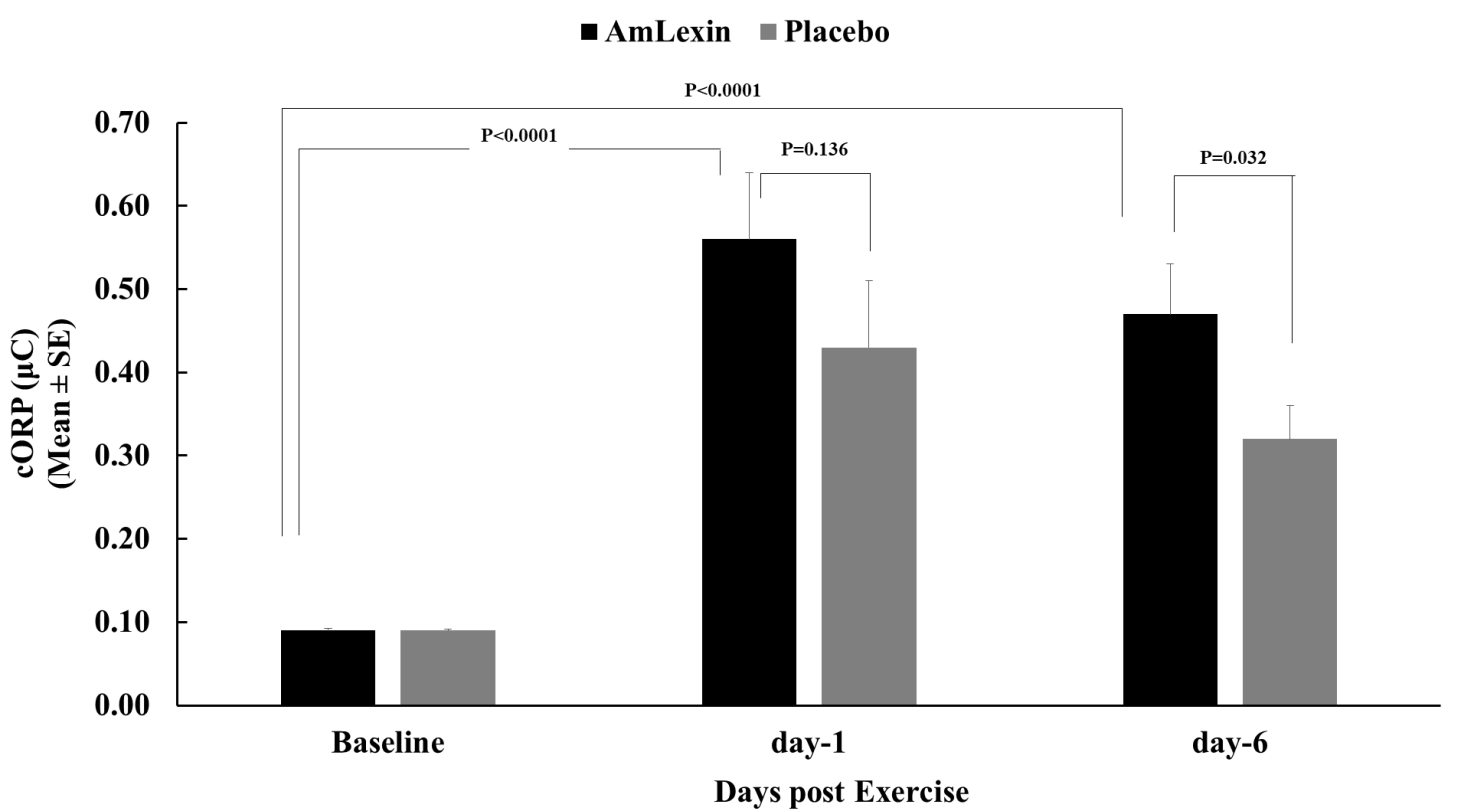
Anti-oxidant capacity of subjects supplemented with AmLexin assessed using the RedoxSYS diagnostic system. Data depicted for capacity oxidation-reduction potential (cORP). Higher cORP corresponds to greater body anti-oxidant reserve.

In the current study, there was clear evidence favoring the beneficial effects of AmLexin in terms of oxidative stress (as measured by sORP) and anti-oxidant reserves (as measured by cORP). Following the half-marathon, there were 29.2% and 29.9% reductions in plasma sORP values at days 1 and 6, respectively, from the baseline for subjects in the AmLexin group. These reductions in Plasma sORP values were lower in magnitude for subjects in the placebo group, with 20.8% and 24.1% reductions on days 1 and 6, respectively (Fig. 10). When these values were compared between groups, there were 9.1% and 6.2% reductions in sORP values for the AmLexin group on days 1 and 6, respectively. The reduction observed on day 6 was statistically significant compared to the placebo group (P < 0.05). Similarly, there were marked increases in the level of plasma cORP for both the treatment groups following the half-marathon (Fig. 11). When compared to the baseline, subjects showed 558.8% and 452.9% increases in plasma cORP for the AmLexin group and 383.5% and 251.6% increases for the placebo group on days 1 and 6, respectively. However, when the values were compared to the placebo group, subjects in the AmLexin group experienced a 27.5% greater increase in plasma cORP on day 1, and a 46.3% greater increase on day 6. The increase on day 6 was statistically significant compared to the placebo group (P < 0.03).

Interestingly, there were suggestive correlations between sORP and cORP in both study groups. While there were negative correlations at day 1 (r = −0.37) and day 6 (r = −0.21) in subjects in the AmLexin group, the correlations were positive for the placebo group both on day 1 (r = 0.18) and day 6 (r = 0.12). These data are indications for significant changes occurring in the opposite direction for subjects in the AmLexin group. In other words, because of AmLexin supplementation, there was enough reduction in oxidative stress while also increasing the antioxidant capacity of individuals.

## DISCUSSION

In the present study, a standardized blend from the heartwood of Acacia catechu and root bark of *Morus alba *(referred to as AmLexin) has been tested for its effect on delayed onset muscle soreness. This was done in a randomized double-blind placebo-controlled clinical trial on healthy subjects, following 13.1 miles of running. The active product was administered twice a day orally at 200 mg for a total daily dose of 400 mg/day over 9 weeks. The placebo group received a look-alike at the same dosage for the same duration. Subjects were trained for 8 weeks before the half marathon and were monitored for 1 week after the running. WOMAC was assessed for discomfort, stiffness, and activity of daily living, and VAS for major body muscles such as quadriceps, hamstrings, calves, and knees. Antioxidant activity of the products was also evaluated upon completion of the study. The study lasted for a total of 9 weeks. Data depicted here showed that subjects in the AmLexin group experienced statistically significant improvement in discomfort, ADL, and total WOMAC compared to the placebo group. There were significant reductions in muscle soreness for the AmLexin group following the half-marathon, with reduced oxidative stress. 

Delayed onset muscle soreness is characterized as a grade 1 muscle strain injury that occurs as a result of unaccustomed exercise, strenuous physical activity, or high-intensity exercise involving eccentric muscle contraction. This includes resistance training, downhill running, cycling, or stepping^4^. It is known to decrease muscle strength, causing a profound impact on the ability to conduct subsequent sessions of exercise. This affects the overall performance of athletes and hinders individuals’ normal routine activities of daily living^23^. A marathon is considered an endurance-demanding exercise that could cause a significant increase in oxidative stress, both in elite athletes and individuals new to running. This suggests that this is a good model for exercise-induced oxidative stress. In this class of intense physical exercise, there is always sustained inflammation and oxidative stress, which ultimately lead to muscle injury, fatigue, and DOMS. 

Currently there are unmet demands for DOMS interventions. Through the years, many DOMS management modalities including stretching, massage, cryotherapy, nonsteroidal anti-inflammatory drugs (e.g. ibuprofen), or supplements rich in phytochemicals (e.g. catechin, quercetin, resveratrol, curcumin, gingerol, etc.) have been suggested. While some have shown future potential, many have been found to be ineffective^24-26^. For example, when healthy subjects (n = 7) were supplemented with resveratrol for 7 days at an oral dose of 600 mg/day, no changes were observed in blood C-reactive protein and VAS pain and discomfort scale 24 hours after the marathon, in comparison with the placebo group^24^. Similar outcomes were observed when healthy subjects were supplemented with Omega-3 fatty acids and isoflavons from soy for 5 weeks. These were administered for 30 days before and 1 week during induction of DOMS through eccentric elbow flexion contractions. The authors reported that there were no significant treatment effects between groups for the physical parameters or biomarkers CK, IL-6, TNF-α, and MDA at 48, 72, and 168 hours post-exercise^25^. Donnelly et al. have also reported that ibuprofen is not an appropriate treatment for delayed onset muscle soreness and damage^26^. One possible reason for this could be the drug’s lack of antioxidant properties. Therefore, an appropriate selection of interventions for DOMS should encompass both antioxidant and anti-inflammatory properties.

The anti-inflammatory and antioxidant activity of AmLexin has been reported both separately and in combination. Previously, AmLexin was tested in subjects with osteoarthritis in the knee, in comparison to active and placebo-controlled randomized clinical trials. Kalman and Hewlings have reported improvements in discomfort, stiffness, and activities of daily living measured by the WOMAC questionnaire and VAS (pain/discomfort) within all groups. There was a significant difference between the changes of uCTX-II for AmLexin and placebo groups after 12 weeks of study. Their data showed that early intervention with AmLexin, aimed at reducing bone and cartilage degradation through reported inhibition of catabolic proinflammatory pathways, might assist in preventing joint cartilage damage^27^. Similarly, Yimam et al. have also reported the anti-inflammatory activities of AmLexin in in vivo and in vitro models with significant implication in DOMS^19^. Recently, we tested the effect of AmLexin on key proinflammatory cytokines such as IL-1β, Il-6 and TNF-α, and documented significant anti-inflammatory activity (manuscript submitted to Nutrients journal). The improved symptom relief observed in this clinical trial could be due to the direct benefits of the anti-inflammatory properties of AmLexin. 

Oxidative stress (reflected in excessive production of ROS) is an imbalance between the production of free radicals and the inherent capacity of the body to counteract or neutralize their harmful effects. Unchecked, it will have detrimental consequences in performance or wellness. For example, excessive exercise (e.g. a marathon) is known to induce production of ROS, resulting in oxidative stress that may cause muscle damage, fatigue, oxidative damage, production of proinflammatory cytokines, and immune dysfunction. Previously, it has been shown that exercise utilizing large muscle groups, such as running, induces oxidative stress in correlation with increases in plasma levels of IL-6^28^. AmLexin has been shown to reduce these factors significantly. The significant reductions in discomfort observed in the major muscles and body parts such as the calves, quadriceps, hamstrings, and knees could be true reflections of the antioxidant and anti-inflammatory activity of AmLexin. 

Substantiating our findings, there is evidence that supports the ability of catechin to prevent exercise-induced muscle damage that could affect physical performances of athletes and/or daily activities of living. For instance, senescence-accelerated animals given daily treatment of catechin for 8 weeks have showed decreased loss of muscle force, exercise-induced muscle damage, and oxidative stress biomarkers such as creatine kinase, acetate dehydrogenase and malondialdehyde, while increasing activity of glutathione reductase. It was also found that long-term use of catechin reduced mRNA expression of TNF-α and monocyte chemoattractant protein-1 from the gastrocnemius muscle, possibly through the inhibition of NF-κB activation^29^. Clinically, chronic consumption of a polyphenolic blend containing catechins and theaflavins for 13 weeks improved antioxidant status. This was measured by the ferric-reducing ability of plasma, reduced markers of muscle stress (such as cortisol and creatine phosphokinase), and the promotion of strength recovery post-exercise^30^. Additionally, increase in the daily energy expenditure^31^, increased VO2 max ^32^, and reduced plasma creatinine kinase^33^ are some of the major activities of catechin, with beneficial applications in exercise and DOMS management. Hence, the significant clinical improvements observed in the current clinical trial could be partially due to the contribution of catechin in AmLexin. Both medicinal plants standardized in AmLexin have traditionally been used for various human ailments with a long history of safe human consumption, making them viable natural alternative for DOMS supplementation.

In summary, exercise can trigger a cascade of inflammatory and oxidative stress-related events, leading to delayed onset muscle soreness. Data depicted here provides noteworthy evidence for the use of antioxidant and anti-inflammatory dietary supplementations such as AmLexin) before or during exercise, in order to minimize exercise-induced oxidative stress and inflammation that could cause discomfort, muscle damage, fatigue, aching pain, inflammation, and ultimately, cellular damage.
